# How Medical Journalism Has Aided the Profession in Texas

**Published:** 1886-04

**Authors:** 


DANIEL'S

### Medical ★ Journal,

PUBLISHED MONTHLY AT

AUSTIN, TEXAS.

F. E. DANIEL, M. D., EDITOR AND PUBLISHER

Subscription Price: Two Dollars per Annum in Advance.

Papers for publication in this Journal should be in the hands of the Editor by the 10th of the month preceding the month in which it is wished to appear; they must be original,—never having appeared in print elsewhere, and must be contributed exclulsively to this Journal.

Contributions to the department of Original Articles are cordially invited from the professiom of Texas, expecially. What is most desired is Clinical reports of cases, essays and short practical articles on any medical topic; also solicited reports of proceedings of societies, clubs, etc., and items of medical news of general interest to the profession, such as notices of appointments of physicians, removals, changes, marriages, deaths, etc.

### EDITORIAL.

### "HOW MEDICAL JOURNALISM HAS AIDED THE PROFESSION" IN TEXAS.

Referring to an editorial on this subject in our last issue, we wish to add, that another and most important outcome of the medical journal is, that it develops talent, and unknown talent, and brings men into prominence, who otherwise would, perhaps, have remained in obscurity all their days.

The chief aim of this journal has been to promote organization of the regular profession of Texas, to the end that it may acquire such power and influence as to be respected, and to be able to procure legislation in the interest of public health. How well we have succeeded so far, is not for us to say. And we do not know, really, how many county and town medical associations have been organized directly and indirectly in consequence of the JOURNAL'S appeals; but certainly many.

We have observed that it is a feature in nearly every one of those local societies to appoint an essayist for each meeting, whose duty it is, is to prepare and read an original paper on some medical subject at next meeting. The best of these essays find their way into print, through the home journal or some other publication, and thus obtain currency amongst the profession in and out of the State. Some of them have, (as stated in our last issue), attracted considerable attention abroad. The authors, many of whom never before put pen to paper for such purpose, thus encouraged, have ventured next, to send or read a paper to the State Association—where it is embodied in the transactions of that society. Indeed, the bulk of the contents of the Transactions of the Texas State Medical Association is made up of such papers—contributed generally to some section by men who had never, ten years ago, written a line; and in late years those papers haye recieved much favorable notice at the hands of the profession. This fact has caused the profession of the outside world to reverse the opinion expressed only a few years ago by the *London Lancet*, that "no good (professionally) can come out of Texas." Moreover, some of our more experienced writers have read papers at the American Medical Association, which have been well received. In this way, then, as well as others mentioned last month, the publication of a journal very effectually aids the medical profession. In Texas, in addition, it is a MEDICAL NEWS medium, and especially for the dissimination of the doings of our many medical societies. It is the duty of the profession to sustain their home journal by every means in their power.

